# 

**Published:** 1895-09

**Authors:** 


*The following donations have been received since the publication of the list in June:*

*August 31st, 1895.*

| | |
|---|---|
| R. E. Burges, M.D. (1) ... ... ... ... ... ... ... | 30 volumes. |
| L. M. Griffiths (2) ... ... ... ... ... ... ... ... | 2 ,, |
| Arthur B. Prowse, M.D. (3) ... ... ... ... ... ... | 4 ,, |
| R. Shingleton Smith, M.D. (4) ... ... ... ... ... | 4 ,, |
| The Staff of Westminster Hospital (5)... ... ... | 7 ,, |

Unbound periodicals have been received from L. M. Griffiths, the Sanitary Publishing Company, and Dr. R. Shingleton Smith.

### SEVENTEENTH LIST OF BOOKS.

The titles of books mentioned in previous lists are not repeated.

The figures in brackets refer to the figures after the names of the donors, and show by whom the volumes were presented. The books to which no such figures are attached have either been bought from the Library Fund or received through the *Journal*.

| | | | | |
|---|---|---|---|---|
| **Banks, W. M.** ... | *The Gentle Doctor—Physic and Letters* ... ... ... | (3) | | 1893 |
| **Barclay, A. W.**... | *Medical Diagnosis* ... ... ... ... ... ... | (1) | 3rd Ed. | 1870 |

**Barnard and G. B. Stocker, W.** *Medical Partnerships, etc.* ... ... ... 1895
**Blundell, J.** ... *The Principles and Practice of Obstetricy.* (Ed. by T. Castle) ... ... ... ... ... ... ... ... ... ... (1) 1834
**Browne, L.**... ... *Diphtheria and its Associates* ... ... ... ... ... ... ... 1895

**Carpenter, W. B.** *The Microscope* ... ... ... ... ... ... ... (1) 6th Ed. 1881

**Cleland, J.** ... ... *Animal Physiology* ... ... ... ... ... ... ... ... (1) 1874

**Cook, Lady**... ... *On the Evils of Society and their Remedies.* 2nd Series 1895

**Erskine, J.**... ... *The Causes of the Neglect of Suppurative Ear-Disease* 1895

**Fernie, W. T.** ... *Herbal Simples* ... ... ... ... ... ... ... ... ... ... 1895

**Gant, F. J.** ... *The Irritable Bladder* ... ... ... ... ... (1) 3rd Ed. 1872

**Gillies, H. C.** ... *Counter-Irritation* ... ... ... ... ... ... ... ... ... 1895

**Gimlette, J. D.**... *Myxœdema and the Thyroid Gland* ... ... ... ... ... ... 1895

**Griffith, J. P. C.** *The Care of the Baby* ... ... ... ... ... ... ... ... 1895

**Hansen and C. Looft, G. A.** *Leprosy.* (Tr. by N. Walker) ... ... 1895
**Harris, T.** ... ... *Indurative Mediastino-Pericarditis* ... ... ... ... ... ... 1895
**Harrison, R.** ... *Some Points in the Practice of Lithotrity or Litholapaxy* 1895
**Hart, E.** ... ... *The Truth about Vaccination* ... ... ... ... 2nd Ed. 1895

**Kingston, W. B.**... *Claret* ... ... ... ... ... ... ... ... ... ... ... ... 1895

**Kirkes, W. S.** ... *Hand-book of Physiology.* 9th Ed. (Ed. by W. M. Baker) ... ... ... ... ... ... ... ... ... ... (1) 1876

*Kissingen Spa* ... ... ... ... ... ... ... ... ... ... ... ... ... ... ... (3) [N.D.]

**Laycock, T.** ... *Mind and Brain.* 2 vols. ... ... ... ... ... (1) 1860

**Lee, R.** ... ... *Clinical Midwifery* ... ... ... ... ... ... (1) 2nd Ed. 1848

**Lewis, H. L.** .. *Medical Electricity* ... ... ... ... .. ... 2nd Ed. 1895

**Lewis, P. G.** ... *Early Scoliosis* ... ... ... ... ... ... ... ... ... ... 1895

**Looft, G. A. Hansen and C.** *Leprosy.* (Tr. by N. Walker) ... ... ... 1895

**Neligan, [J. M.]** *Medicines.* 7th Ed. (Ed. by R. Macnamara)... (1) 1867
**Nicolaysen, J.** ... *Studier over Ileus* ... ... ... ... ... ... ... ... ... 1895
**Nocard, E.**... ... *The Animal Tuberculoses and their Relation to Human Tuberculosis.* (Tr. by H. Scurfield) ... ... ... 1895

**Parkes, L. C.** ... *Hygiene and Public Health* ... ... ... ... 4th Ed. 1895

**Pereira, [J.]** ... *Materia Medica and Therapeutics.* (Ed. by F. J. Farre, R. Bentley, and R. Warington) ... ... ... (1) 1865

**Prentice, C.** ... *The Eye in its Relation to Health* ... ... ... ... ... 1895

**Pye-Smith, P. H.** *The Ætiology of Disease, etc.* ... ... ... ... ... ... 1895

**Quain, [J.]**... ... *Elements of Anatomy.* 10th Ed. (Ed. by E. A. Schäfer and G. D. Thane.) ... ... Vol. III., Part II. 1895

**Shuttleworth, G. E.** *Mentally-deficient Children* ... ... ... ... ... ... 1895

**Simon, J.** ... ... *Public Health Reports.* 2 vols. (Ed. by E. Seaton) (1) 1887

**Snow, H.** ... ... *Radical Cure in Cancer, and other Reprinted Papers* ... 1895

**Squire, P.** ... ... *Companion to the British Pharmacopœia.* 14th Ed. (Ed. by P. W. and A. H. Squire) ... ... ... (1) 1886

**Stocker, W. Barnard and G. B.** *Medical Partnerships, etc.* ... ... ... 1895

**Stokes, W.**... ... *Diseases of the Chest*... ... ... ... ... Part I. (1) 1837

**Thompson, Sir H.** *Diseases of the Urinary Organs* ... ... ... ... (1) 1868

### TRANSACTIONS, REPORTS, JOURNALS, &c.

American Dermatological Association, Transactions of the ... ... ... 1894
American Journal of Obstetrics, The ... ... ... ... ... Vol. XXXI. 1895
American Journal of Ophthalmology, The ... ... ... ... ... Vol. XI. 1894
American Journal of the Medical Sciences, The... ... ... Vol. CIX. 1895
Archives de Neurologie ... ... ... ... ... ... ... ...Tome XXIX. 1895
Asclepiad, The ... ... ... ... ... ... ... ... (2) Vols. IV. 1887, VI. 1889
Australian Medical Journal, The ... ... ... ... Vols. XV., XVI. 1893-94
Bäder-Almanach... ... ... ... ... ... ... ... ... ... ... ... ... (3) [1895]
Birmingham Medical Review, The ... ... ... ... Vol. XXXVII. 1895
Boston Medical and Surgical Journal, The... ... ... Vol. CXXXII. 1895
Bristol Directory ... ... ... ... ... ... ... ... (4) 1876, 1878, 1887, 1892
Bristol Health Report, 1894 ... ... ... ... ... ... ... ... ... ... ... 1895
British Gynæcological Journal, The ... ... ... ... ... ... Vol. X. 1894
British Medical Journal, The ... ... ... ... ... ... ... Vol. I. for 1895
Bulletins et Mémoires de la Société de Médecine et de Chirurgie pratiques de Paris ... ... ... ... ... ... ... ... ... ... ... ... ... 1894
Centralblatt für innere Medicin ... ... ... ... ... ... ... ... ... 1894
Chicago, Annual Report of the Department of Health of, 1894 ... ... 1895
Cincinnati Lancet-Clinic, The ... ... ... ... ... Vol. LXXIII. 1895
Dictionary of Bathing Places, Bradshaw's ... ... ... ... ... ... ... 1895
Dublin Journal of Medical Science, The (1) Vols. LXXXIII., LXXXIV., 1887; XCIX. 1895
Edinburgh Medical Journal ... ... ... ... ... ... ... ...Vol. XL. 1895
Edinburgh Obstetrical Society, The Transactions of the Vol. XIX. 1894
Hospital and Charities Annual, Burdett's ... ... ... ... ... ... ... 1895
Hospitals—

- Edinburgh Hospital Reports ... ... ... ... ... ... ...Vol. III. 1895
- Johns Hopkins Hospital Reports, The ... ... Vol. IV., No. 7-8 1895
- London Temperance Hospital. Report upon the Surgical and Ophthalmic Cases admitted during the year 1894
- Westminster Hospital Reports, The... ... (5) Vols. I.—VII. 1885-91

Indian Medico-Chirurgical Review, The ... ... ... ... .. Vol. II. 1895
Intercolonial Quarterly Journal of Medicine and Surgery ... Vol. I. 1895
Lancet, The... ... ... ... ... ... ... ... ... ... ... Vol. I. for 1895
Medical News, The ... ... ... ... ... ... ... ... .. Vol. LXVI. 1895
Medical Press and Circular, The ... ... ... ... ... ... Vol. CX. 1895
Medical Record ... ... ... ... ... ... ... ... ... ... ...Vol. XLVII. 1895
New York Academy of Medicine, Transactions of the ... ... Vol. X. 1894
New York Medical Journal, The ... ... ... ... ... ... Vol. LXI. 1895
Practitioner, The ... ... ... . ... ... ... ... ... [Vol. LIV.] 1895
Progrès Médical, Le ... ... ... ... ... ... ... ... 3e Sér. Tom. I. 1895
Provincial Medical Journal, The ... ... ... ... (1) Vols. V.—VIII. 1886-89
Retrospect of Medicine, Braithwaite's ... ... ... ... ... Vol. CXI. 1895
Small-pox in Bristol during the Years 1893-4, Report on... ... .. ... 1895
Whitaker's Almanack ... ... ... ... ... ... ... ... ... ... ... (3) 1893
Year-Book of Pharmacy ... ... ... ... ... ... ... ... ... ... ... (1) 1878
Year-Book of Treatment, The... ... ... ... ... ... ... ... ... ... 1895

### The William F. Jenks Memorial Prize.

This Prize, the competition for which we announced in June, 1894, has been awarded to A. Brothers, M.D., 162 Madison Street, New York, for the best essay on "Infant Mortality During Labour, and its Prevention."

### Local Medical Notes.

### BRISTOL HEALTH REPORTS.

The Medical Officer of Health and his assistants are to be congratulated on the very satisfactory Reports that have just been published and distributed, and the public of our city should be made acquainted with the fact that, though our sanitary rate may vie with that of most towns for size, we can boast of being not only one of the healthiest towns in England but in the world. Dr. Davies tells us that with 3888 deaths the general death-rate for 1894 is 17.16 per 1000 living, the lowest rate yet recorded for the city; but the ugly fact is mentioned that of these deaths 948 were of infants under one year, giving an infant death rate of 48.31 (*i.e.*, proportion to 1000 births). During 1894, 6393 births were registered (of which 2.9 per cent. were illegitimate) giving a birth rate of 28.81, continuing the decrease noticed with three exceptions since 1881.

Attention is called to the falling off in the number of cases of diphtheria—118 cases compared with 142 in 1893—and to the very high mortality from that disease; viz., 39.0 per cent. The number of cases of erysipelas (154 with 8 deaths), of scarlet fever (485 with 10 deaths), and of enteric fever (90 with 21 deaths), show a distinct falling off, while there is a slight increase in the number of fatal cases of measles and whooping cough. No cases of typhus or cholera occurred during the year.

Coming to the important question of vaccination, we find that in the Bristol Union in 1893, out of 1542 children 1186 were successfully vaccinated, a percentage on the total births of 76.91, a slight improvement on the returns of 1892, and comparing favourably with the returns for England and Wales for 1891. We cannot help thinking, however, that 13 insusceptible children is a large number. In the Barton Regis Union 6502 children were successfully vaccinated out of 8918 born between Jan. 1st, 1893, and June 30th, 1894, a percentage to the total births of 72.0. Here again we notice a large number—45—returned as insusceptible. We should like to know in how many of these cases there had been arm-to-arm vaccination.

On turning to the Supplementary Report on small-pox during the years 1893 and 1894 we find some interesting and instructive statistics and information on the introduction and spread of the disease. During these two years small-pox was introduced eleven times, 360 persons being attacked, 36 of whom died. The space at our disposal does not allow of a thorough epitome of the Report, which must be referred to for further information. We should like, however, to draw attention to the following facts, which we think are worthy of consideration :

(1) That the heaviest mortality occurred in the unvaccinated (41 cases with 11 deaths, or 26.8 per cent.), while in the patients—60—with four marks, the mortality—2—was 3.3 per cent., and the disease was mild in all but six cases. (2) That among the staff of 62 persons, composed of doctors, nurses, inspectors, labourers, and ambulance staff, all fully exposed to infection, only three took the disease, one terminating fatally. Of the two cases which recovered one had not been revaccinated, the other had been fourteen years before, while the fatal case occurred in a washerwoman, unrevaccinated for twenty years, who had been in contact with the disease for years and was consequently considered secure. Dr. Davies justly remarks that the protection of vaccination, though a real one, has a limit of time. There are many more interesting facts to be gathered from the Report, which will well repay a careful perusal.

In consequence of the prevalence of cholera upon the Continent during 1893, it was necessary that continued vigilance should be exercised during the spring, summer, and autumn months of 1894, when its recrudescence was most to be feared. The Port Sanitary District Report embodies an account of all the precautions which were taken, and which were eminently successful.

Dr. Davies calls attention to the inadequate means at his disposal for the proper disinfection of articles removed from infected houses. The disinfector—the Washington-Lyons—meets all requirements, but the receiving and distributing rooms require enlarging and remodelling. We hope that the Sanitary Committee will see their way to this outlay without any increase of the present rates.

The Meteorological Report by Mr. Rintoul, of Clifton College, is of considerable interest and displays great care in observation and compilation. It may interest our readers to know that 5.18 inches of rain fell between Nov. 8th and 15th, 1894, which we believe is a record.

### UNIVERSITY COLLEGE, BRISTOL.

List of students who have recently passed examinations :—

**University of London.**—*Preliminary Scientific* :—Entire Examination. First Division—R. G. Johnson. *Chemistry and Experimental Physics*—J. D. C. Callcott, A. Coleridge*. *Biology*—F. H. Pickin.

Intermediate Examination in Medicine, July, 1895 :—Entire Examination. First Division—W. R. Battye. Excluding Physiology. Second Division—H. T. S. Aveline, A. B. Cridland, W. S. V. Stock.

**Examining Board in England by the Royal Colleges of Physicians and Surgeons.**—First Examination. Part I. :—*Chemistry and Physics*—A. Coleridge, R. M. McQueen. *Practical Pharmacy*—W. E. H. Hancock, C. F. Walters. *Elementary Biology*—J. B. S. D'Aguilar, F. B. Hughes, C. W. W. James. *Elementary Anatomy*—J. C. Clayton, H. Hemstead.

**Royal College of Surgeons.**—June 13: *Licentiate in Dental Surgery*—F. Little. July 1: Second Examination. *Anatomy and Physiology*—R. J. Phillips, E. M. Pearce, F. Cox. *Physiology only*—C. M. Mitchell, F. C. Morgan.

**Society of Apothecaries.**—Pass List, August, 1895: *Surgery*—H. S. Maw, *Medicine*, *Forensic Medicine and Midwifery*—H. S. Maw.

* Completes Examination.

